# Image Generation Using Bidirectional Integral Features for Face Recognition with a Single Sample per Person

**DOI:** 10.1371/journal.pone.0138859

**Published:** 2015-09-28

**Authors:** Yonggeol Lee, Minsik Lee, Sang-Il Choi

**Affiliations:** 1 Department of Computer Science and Engineering, Dankook University, 126, Jukjeon-dong, Suji-gu, Yongin-si, Gyeonggi-do, 448–701, Korea; 2 Division of Electrical Engineering, Hanyang University, 55 Hanyangdaehak-ro, Sangnok-gu, Ansan-si, Gyeonggi-do, 426–791, Korea; Ulm University, GERMANY

## Abstract

In face recognition, most appearance-based methods require several images of each person to construct the feature space for recognition. However, in the real world it is difficult to collect multiple images per person, and in many cases there is only a single sample per person (SSPP). In this paper, we propose a method to generate new images with various illuminations from a single image taken under frontal illumination. Motivated by the integral image, which was developed for face detection, we extract the bidirectional integral feature (BIF) to obtain the characteristics of the illumination condition at the time of the picture being taken. The experimental results for various face databases show that the proposed method results in improved recognition performance under illumination variation.

## Introduction

Face recognition is used to identify individuals from facial images by using a face database labeled with people’s identities [1]. Compared to other types of biometric recognition, face recognition is less invasive and does not require a subject to be in proximity to or in contact with a sensor, which makes it widely applicable in areas including user identification, e-commerce, access control, surveillance, and human-computer interaction [1]. For this reason, face recognition has received extensive attention from many researchers over the last two decades.

The motivation for using appearance-based methods, the most widely adopted approach in the face recognition field [2–6], is its ability to construct a small-scale, low-dimensional feature subspace that maintains the intrinsic characteristics of the original face samples by using supervised, semi-supervised, or unsupervised learning. Beginning from the most basic schemes such as the Eigenface [2] and PCA+LDA [3] methods have continuously evolved to produce other methods including Discriminant Common Vector (DCV) [4], Direct Linear Discriminant Analysis (Direct LDA) [5] and Eigenfeature Regularization and Extraction (ERE) [7], and Marginal Fisher Analysis (MFA) [8]. These methods transform each image into a vector form, and then extract appropriate features from various kinds of covariance matrices based on a statistical analysis. The methods based on discriminant analysis such as the variants of LDA (linear discriminant analysis) seek the linear transformation that minimizes within-class variation while simultaneously maximizing the between-class variation; although, this is only effective when the within-class variance is small and the between-class variance is large. It is important to note that numerous problems still need to be overcome to develop a robust face recognition system.

In a real-world setting, facial-data acquisition can occur in many different environments, so a sufficient number of facial images are therefore needed to construct a face recognition system that is reliable under various conditions. Numerous face recognition methods have been proposed for face recognition under the assumption that a number of images are accessible for each individual, but in fact, a much smaller number of training images can be acquired in most real-world applications [9]. To use a specific example, large-scale identification applications including law enforcement and passport identification typically use databases that contain a single training sample per person (SSPP). Additionally, due to the exorbitant monetary cost of capturing additional samples, further training images are rarely added to an individual’s profile; furthermore, for an image collection—in which several training samples of an individual are accessible—to be useful, the images need to have been taken under a variety of conditions to account for different variations [9].

The small number of training samples for each person raises several problems for appearance-based face recognition systems. If the feature dimension of the face samples is larger than the number of training images, it is not possible to apply LDA without hindrance because the within-class scatter matrix develops into a singular form—this issue is known as the small sample size (SSS) problem. The number of training samples per person continues to exert a major influence on the functioning of appearance-based methods in face recognition, even though the variants of the LDA method, such as PCA+LDA, DCV, and Direct LDA, were considered solutions to the SSS problem. Of particular relevance here is an understanding that a considerably lower number of training samples per person relative to the feature dimension equates to an inability to both accurately estimate the within-class variance of the LDA and make use of distinguishing data [10].

To address the problems that stem from the small number of training samples per person, several methods have been introduced. In [11–15], new representational methods for mining more information from a single image were proposed. In [11], the representational oriented component analysis (ROCA) was presented. This method applies several linear and non-linear filters to each gallery and for production of its 150 representations. The method in [13] uses the singular value decomposition (SVD) perturbation to extract the greatest amount of information possible from a single training image. In the E(PC)^2^A2+ method [14], new images are generated by linearly combining the original image and its corresponding 1/2-, first-, and second-order projected images. In [15], many samples are synthesized by using real images (sets of two) and their weighted combination. However, since the images generated by the above methods are highly correlated, the new images cannot be considered as independent training images [16].

Some methods have proposed to generate virtual face images to enlarge the training set. In [17], the virtual images were produced by using the symmetry transform for intra-class and the linear combination for inter-class, while in proposal [18], the symmetrical PCA method uses even and odd symmetrical image sets. The method in [19] also uses the symmetrical structure of a face to generate new training samples. However, while most of the above methods mainly focused on enlarging the training set, they did not consider the different variations such as illumination variation that are likely to occur in uncontrolled conditions.

In this paper, we propose a novel approach to generate new face images from a single training image to solve the SSPP problem. We first propose the bidirectional integral features (BIF) based on the idea of the integral image [20]. Since the value at (*x*, *y*) of the integral image is the sum of pixels above and to the left of (*x*, *y*) in the original image, its first-order derivatives represent the distribution of gray-level intensities in the sub-region, i.e., the first-order derivatives in the corresponding region of the integral image are small for the dark region of the original image, while those for the bright region are large. We defined two kinds of integral images, which are the left and right integral images, depending on the direction of the light source. The values of the left and right integral images were obtained by calculating the sum from the left-top to the right-bottom, and from the right-top to the left-bottom, respectively. Then, we extracted BIF by normalizing each integral image for values ranging between 0 and 1.

In terms of shape, human faces are typically similar, consisting of two eyes, one nose, and one mouth. Each of these facial components casts shadows upon the face, of which the form is reliant upon the location of the light source. When the light source is located at one side of the face, the shadows occur on the opposite side of the face. As the light source moves away from the frontal direction, the attached and cast shadows become severe and large. Since the region with shadows leads to small BIF, we extract illumination-variation information from these BIF. Based on the illumination information, we generate several new face images that are similar to the images taken under various illuminations, but are derived from only a single image taken under a frontal illumination.

Our proposed method for circumventing the SSPP issue is advantageous on a number of fronts compared with comparable algorithms. Once the BIF for the pre-defined light direction categories is defined, we can simply generate new images from a face image for each of the pre-defined categories. As a result, we not only solve the SSS problem, but can also effectively deal with face recognition under illumination variation. Additionally, the proposed method is not reliant upon the choice of a particular appearance-based face recognition algorithm, and a single training sample per person is sufficient to improve face recognition performance when there are illumination variations, as shown in the experimental results.

The remainder of this paper is organized as follows. In the next section, we provide preliminaries for the proposed method. Then, we present the BIF and how they can be used to generate new face images with shadows. Finally, the experimental results are described and the conclusion follows.

## Preliminaries to the SSPP Problem in Appearance Based Methods

The SSPP problem, which is an extreme case of the SSS problem in classification, is defined as a key problem of face recognition technology, as there is only a single sample for each person. In this section, we provide a brief overview of the feature extraction methods of appearance-based face recognition methods, and explain how the SSPP problem influences these methods. LDA, which was originally applied in the supervised learning field, has been popularly adopted for its capacity to pare down the dimensions of a spatial context for easier handling [21, 22]. Let us consider a set of *N* samples, each of which belongs to one of *N*_*c*_ subjects or classes. Each sample **x** ∈ **R** ^*n*^ can be represented as a point in the *n*-dimensional vector space. Let **x**_*ij*_ denote the *j*th sample belonging to the *i*th class. The *i*th class consists of *N*_*i*_ samples, and *N* = ∑_*i*_ *N*_*i*_. The LDA method finds the optimal projection matrix in accordance with Fisher’s criterion to maximize the ratio of the between-class scatter matrix (*S*_*B*_) and the within-class scatter matrix (*S*_*W*_).

$$\begin{array}{c} \begin{array}{cc} W & =\arg\max_{W}\frac{|W^{T}S_{B}W|}{|W^{T}S_{W}W|},\\ S_{B} & =\frac{1}{N}\sum_{i=1}^{N_{c}}\left(m_{i}-m\right){\left(m_{i}-m\right)}^{T},\\ S_{W} & =\frac{1}{N}\sum_{i=1}^{N_{c}}\sum_{j=1}^{N_{i}}\left(x_{ij}-m_{i}\right){\left(x_{ij}-m_{i}\right)}^{T}, \end{array} \end{array}$$ (1)

where **m** is the mean of all the samples and **m**_*i*_ is the mean of the samples belonging to class *i*. The column vectors of *W* = [**w**_1_, .., **w**_*N*_*c*_−1_] are the generalized eigenvectors associated with the generalized eigenvalues satisfying

$$\begin{array}{c} S_{B}w_{k}=\lambda_{k}S_{W}w_{k}, \end{array}$$ (2)

where *k* = 1, .., *N*_*c*_ − 1 [23]. They can be obtained by the simultaneous diagonalization of *S*_*B*_ and *S*_*W*_ if *S*_*W*_ is nonsingular [21].

In face recognition problems, since the dimension of the input space(*n*) is usually much larger than the number of available samples (*N*), *S*_*W*_ becomes singular, resulting in the SSS problem [21]. To avoid the SSS problem, several variants of LDA have been proposed [2–5] including PCA+LDA, Direct LDA, DCV, and ERE. However, even though the SSS problem is solved in terms of computation, some issues regarding the SSPP problem remain outstanding. Firstly, in the case of the SSPP problem, the *S*_*W*_ can not actually be computed. Also, LDA performance is biased toward data that complies with the assumption that normal distribution applies to the samples in each class [24]—this is evidenced by a demonstration in which the full realization of the objective function in Eq 2 corresponds to the full realization of the Euclidean distance that occurs between the class means [25]. Therefore, to effectively overcome the SSPP problem, it is important to secure several images for each class so that the samples in each class have normal distribution.

In subsequent sections, we use the proposed method to demonstrate the improvement in face recognition performance, and show how BIF can be used with the virtual face image-generation method.

## Proposed Method

### Bidirectional Integral Features

The integral image was originally designed to very rapidly compute rectangular features for face detection. The following definition of the integral image states [20]:

$$\begin{array}{c} A\left(x,y\right)=\sum_{x^{\prime }\leq x,y^{\prime }\leq y}I(x^{\prime },y^{\prime }), \end{array}$$ (3)

where the integral image is represented by *A*(*x*, *y*) and the original image is represented by *I*(*x*′, *y*′). In Eq 3, the tally of the pixels above and to the left of (*x*, *y*) forms the integral image at the (*x*, *y*) position. *A*(*x*, *y*) monotonically increases as *x* and *y* increase because all of the pixels of *I*(*x*, *y*) have non-negative values.

**Fig 1:**
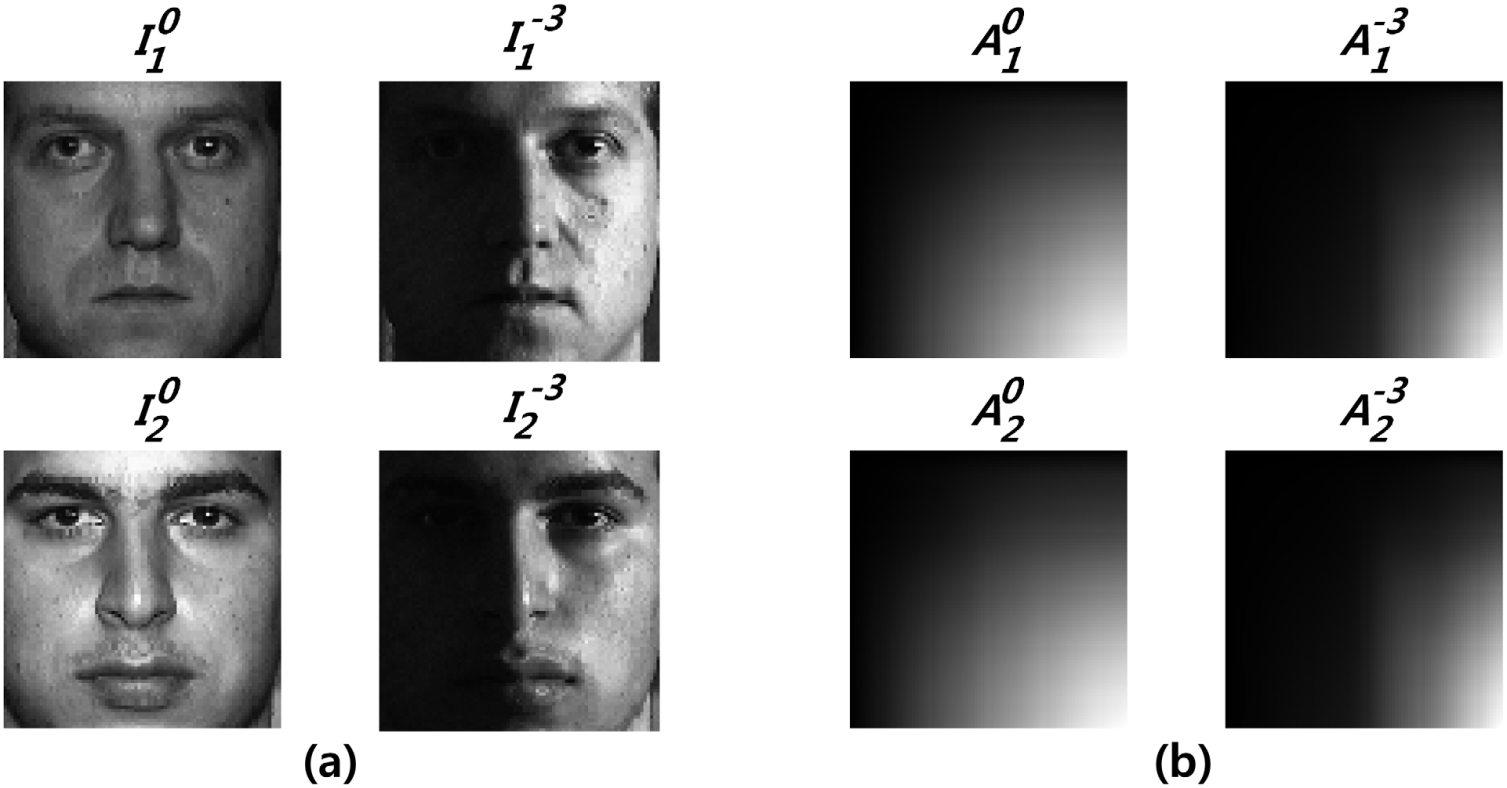
The patterns of integral images. (a) individual images. (b) integral images.

The shape outline of the human face shows an azimuth convex emphasis, so we therefore distinguished the directions of light into *L* categories {*C*_*l*_∣ − *L* ≤ *l* ≤ *L*} (here, *L* = 3), starting at the left side and moving across until the endpoint on the right. For a frontal light source, *C*_0_ was used. We denoted a face image of the *m*th individual under frontal illumination and right-side illumination as $I_{m}^{l}$, *l* = 0 and $I_{m}^{l}$, *l* = −3, respectively, and the corresponding integral image as $A_{m}^{l}$, *l* = 0 and $A_{m}^{l}$, *l* = −3, respectively. Fig 1 shows the integral images, which were obtained from two face images for two individuals (*m* = 1,2) from the CMU-PIE database [26]; frontal illumination was used for one of the face images and the other was subject to right-side illumination. The images in Fig 1 are scaled to have values ranging between 0 and 255. The patterns of integral images are more dependent on the differences of illumination conditions than the unique features of individuals. As shown in Fig 1, different individuals $A_{1}^{0}$ and $A_{2}^{0}$ are similar to each other, whereas under different illumination conditions, $A_{1}^{0}$ and $A_{1}^{-3}$ are different.

**Fig 2:**
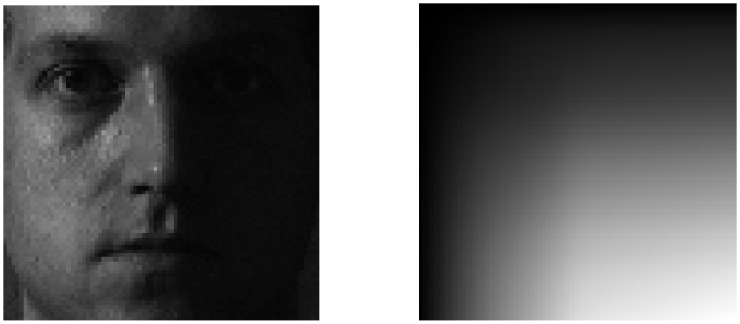
The saturated pattern of integral images from right side illumination.

We characterized the illumination conditions depending on the direction of the light source by using integral images. Meanwhile, in the case of an image under the right-side illumination (Fig 2), since the shadows occur on the right side of the image, where the corresponding pixels have small intensity values, the values of the integral image can be saturated (or flattened with large values) after the middle position of the image. To effectively obtain the characteristics of both left- and right-side illuminations, we defined the left integral image (*B* ^*L*^(*x*, *y*)) and the right integral image (*B* ^*R*^(*x*, *y*)) as follows:

$$\begin{array}{c} \begin{array}{cc} B^{L}\left(x,y\right) & =A(x,y)\\ B^{R}\left(x,y\right) & =\sum_{x^{\prime }\geq x,y^{\prime }\geq y}I(x^{\prime },y^{\prime }). \end{array} \end{array}$$ (4)

We called the above pair of integral images as “bidirectional integral images”. We applied the left integral image (*B* ^*L*^(*x*, *y*)) for categories *C*_−3_ ∼ *C*_−1_ and the right integral image (*B* ^*R*^(*x*, *y*)) for categories *C*_1_ ∼ *C*_3_.

The bidirectional integral images *B* ^*L*^(*x*, *y*) and *B* ^*R*^(*x*, *y*) of one person are insufficient for extracting information about illumination condition because *B* ^*L*^(*x*, *y*) and *B* ^*R*^(*x*, *y*) also contain illumination information, as well as the partially unique features of each individual. To extract the characteristics of the illumination conditions, we need to eliminate the influence of the features that are innate to each individual. For this, we conducted the following few processes. Firstly, to avoid the influence of overall skin tone and race, we normalized the integral images to have values ranging between 0 and 1 as follows:

$$\begin{array}{c} B_{N}^{L}\left(x,y\right)=\frac{B^{L}\left(x,y\right)}{\text{max}(B^{L})},\;\;\;B_{N}^{R}\left(x,y\right)=\frac{B^{R}\left(x,y\right)}{\text{max}(B^{R})}. \end{array}$$ (5)

**Fig 3:**
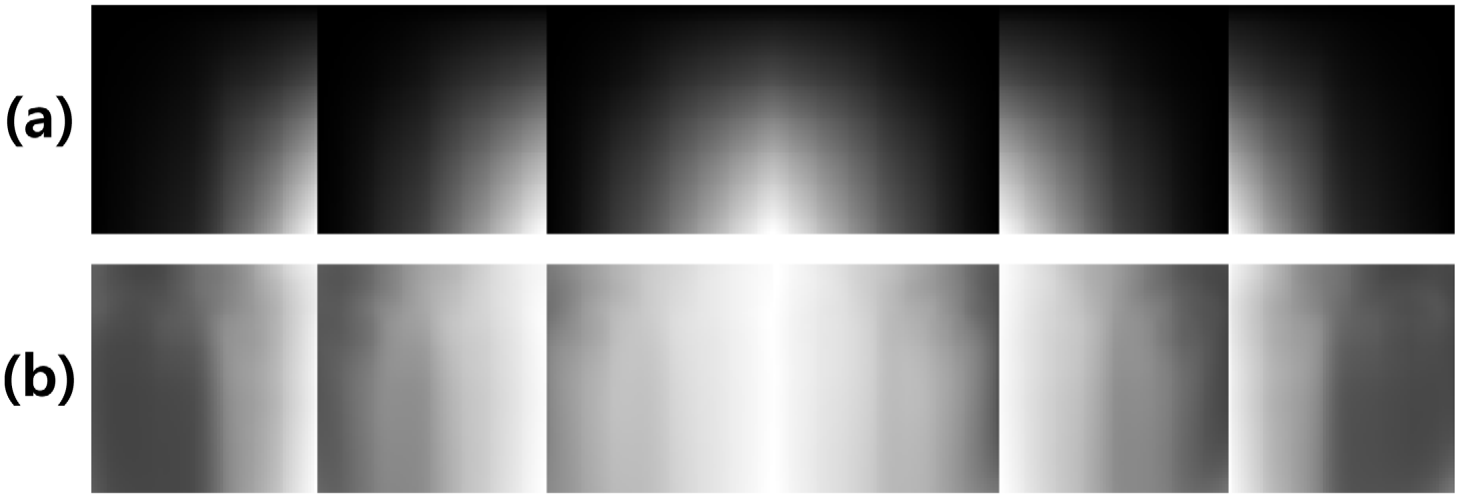
Feature generation. (a) average bidirectional integral images. (b) bidirectional integral features (BIF).

Secondly, we defined the average integral images $B_{l}^{L}\left(x,y\right)$ and $B_{l}^{R}\left(x,y\right)$ for the category *C*_*l*_ as follows:

$$\begin{array}{c} B_{l}^{L}=\frac{1}{M}\sum_{m=1}^{M}B_{N(l,m)}^{L},\;\;\;B_{l}^{R}=\frac{1}{M}\sum_{m=1}^{M}B_{N(l,m)}^{R}, \end{array}$$ (6)

where the subscripts *m*(= 1, 2, .., *M*) denote the *m*th individual in the category *C*_*l*_. Since these average bidirectional integral images represent the general characteristic of the illumination for the category *C*_*l*_, it can be applied without regard to the individuals. The average bidirectional integral images in Fig 3a were made from the images belonging to each category *C*_*l*_ for 40 subjects in the CMU-PIE database. Table 1 shows the face images used in obtaining the average bidirectional integral images for each category *C*_*l*_.

**Table 1:** Classification for illumination of frontal face in CMU-PIE database.

Classes	Flashes	Indices of Images
*C*_−3_	f02, f03, f04	02, 03, 04
*C*_−2_	f05, f10, f18	05, 10, 18
*C*_−1_	f06, f07, f19	06, 07, 19
*C*_0_	f08, f11, f20	08, 11, 20
*C*_1_	f09, f12, f21	09, 12, 21
*C*_2_	f13, f14, f22	13, 14, 22
*C*_3_	f15, f16, f17	15, 16, 17

On the other hand, $B_{l}^{L}$ and $B_{l}^{R}$ contain the overall illumination information for the category *C*_*l*_, which includes not only the distinctive characteristics of *C*_*l*_, but also the information for the ambient illumination (here, frontal illumination). To counteract the effects of the ambient illumination in $B_{l}^{L}$ and $B_{l}^{R}$, we extracted the bidirectional integral features ($F_{l}^{L}$, $F_{l}^{R}$) by dividing $B_{l}^{L}$ and $B_{l}^{R}$ into $B_{0}^{L}$ and $B_{0}^{R}$, respectively, as follows:

$$\begin{array}{c} F_{l}^{L}\left(x,y\right)=\frac{B_{l}^{L}\left(x,y\right)}{B_{0}^{L}\left(x,y\right)},\;\;\;F_{l}^{R}\left(x,y\right)=\frac{B_{l}^{R}\left(x,y\right)}{B_{0}^{R}\left(x,y\right)}. \end{array}$$ (7)

We call the above features BIF, which are used to generate new images in the following section. Fig 3b shows BIF for each category.

### Generation of New Images from a Single Face Image

In face recognition, since the illumination variation is not closely related to the identity of a face, it is akin to static in the way that its hampers the recognition process. We assumed that an original image, which is taken under frontal illumination (an image in *C*_0_), are corrupted through illumination variation to become shadowed face images. We represented the corrupted image (*I*_*Cor*_) apart from the original image (*I*_*Ori*_) by using the following noise model [27]:

$$\begin{array}{c} I_{Cor}\left(x,y\right)=c\left(x,y\right)\cdot I_{Ori}\left(x,y\right)+b\left(x,y\right), \end{array}$$ (8)

where *c*(*x*, *y*) and *b*(*x*, *y*) are contrast and brightness factors at (*x*, *y*), respectively.

In Eq 7, the BIF are the relative illumination characteristics of *C*_*l*_ alongside those in *C*_0_. By representing the changes of contrast from illumination variation as $F_{l}^{L}\left(x,y\right)$ and $F_{l}^{R}\left(x,y\right)$, we generated new face images ($I_{New}^{l}$, *l* ≠ 0), which correspond to *C*_*l*_, from the image under frontal illumination (*I* ^*l*^, *l* = 0), as follows:

$$\begin{array}{c} \begin{array}{cc} I_{New}^{l}\left(x,y\right) & =F_{l}^{L}\left(x,y\right)\cdot I^{0}\left(x,y\right)\;\;\;\text{for}\;\;\;l\in \left\{-3,-2,-1\right\}\\ I_{New}^{l}\left(x,y\right) & =F_{l}^{R}\left(x,y\right)\cdot I^{0}\left(x,y\right)\;\;\;\text{for}\;\;\;l\in \left\{1,2,3\right\}. \end{array} \end{array}$$ (9)

These are the procedural instructions from a summary of our proposed method:

- **Step 1**: Define the categories of light direction in *C*_*l*_.
- **Step 2**: After obtaining the normalized bidirectional integral images ($B_{N(l,m)}^{L}$, $B_{N(l,m)}^{R}$) for each category *C*_*l*_, compute the average integral images ($B_{l}^{L}$, $B_{l}^{R}$).
- **Step 3**: Extract the BIF ($F_{l}^{L}$, $F_{l}^{R}$) by dividing $B_{l}^{L}$ and $B_{l}^{R}$ into $B_{0}^{L}$ and $B_{0}^{R}$, respectively.
- **Step 4**: Generate new face images $I_{New}^{l}$ for each category *C*_*l*_ from *I* ^0^ by using Eq 9.

**Fig 4:**
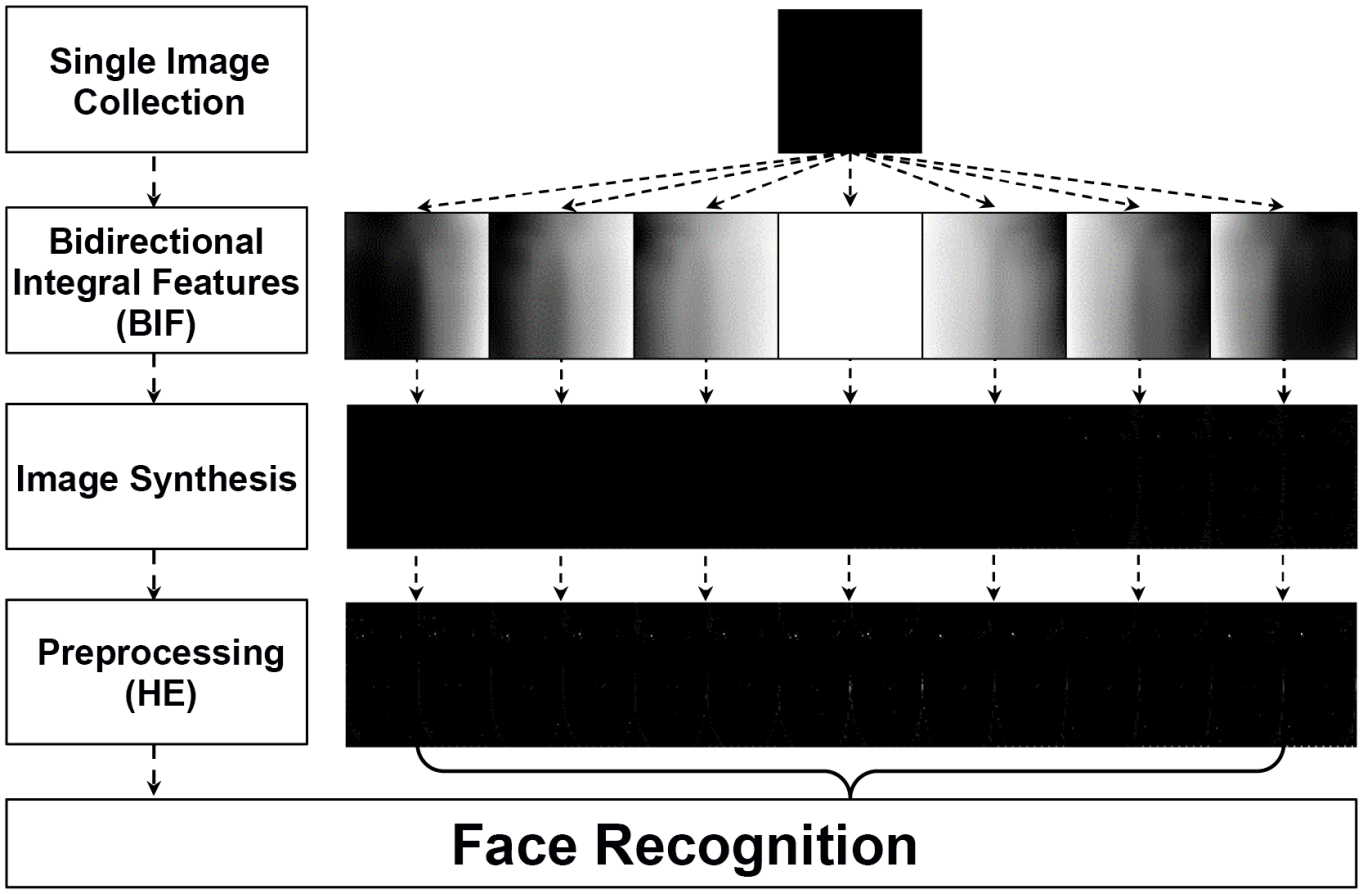
Schematic diagram for proposed method.

Once the BIF are obtained for each category *C*_*l*_, the BIF can be utilized to generate new face images for any individual because the BIF depend solely on the light source direction. Fig 4 shows the overall procedure of the proposed method including the images at each process: a single collected face image (under frontal illumination), BIF for each light category *C*_*l*_, and the generated face images for the category.

## Experimental Results

### Image Generation

To see how well the proposed method generated new images under different illumination variations, we compared the images generated by the proposed method with the raw images from the CMU-PIE database, which are actually taken under different illumination conditions.

**Fig 5:**
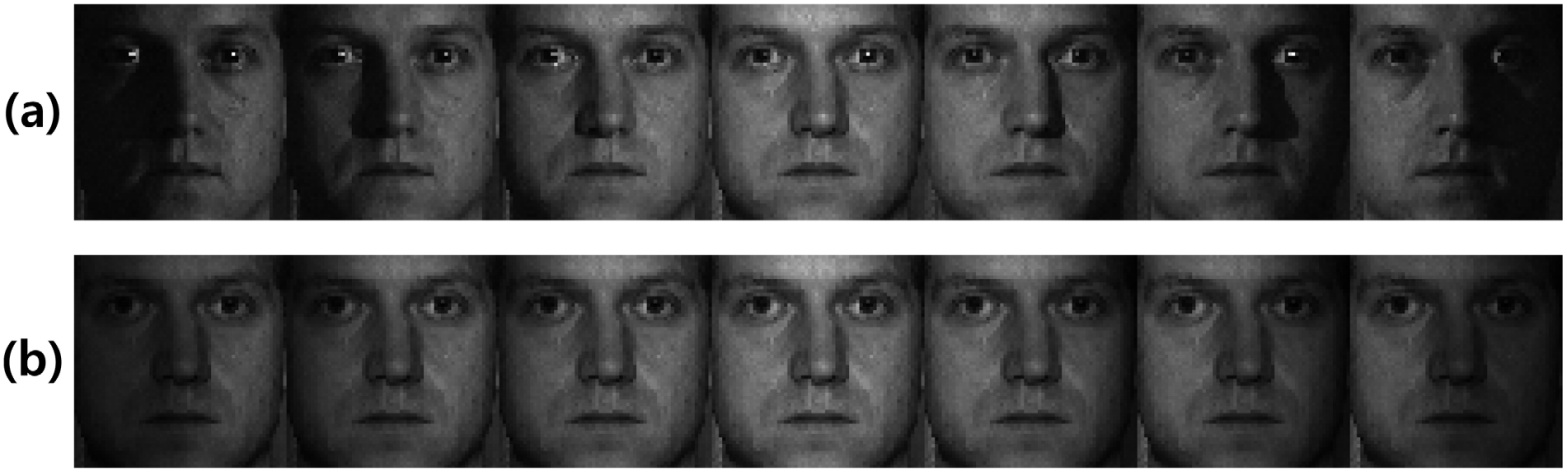
Image generation from a single image(*I* ^0^) by using BIF. (a) images for each light category(*C*_−3_ ∼ *C*_3_). (b) generated images($I_{New}^{-3}∼I_{New}^{3}$).

We selected 40 subjects and 7 images of each subject with different illumination variations (‘27_04′, ‘27_05′, ‘27_06′, ‘27_11′, ‘27_12′, ‘27_14′, and ‘27_15′) for each light category *C*_−3_ ∼ *C*_3_ (Fig 5a) to obtain the BIF (see Fig 3). Fig 5b shows the images generated from a single image with the use of the proposed method. As can be seen in Fig 5a and 5b, the proposed method generated new images for each category *C*_*l*_ as if they were taken under different illumination conditions.

**Fig 6:**
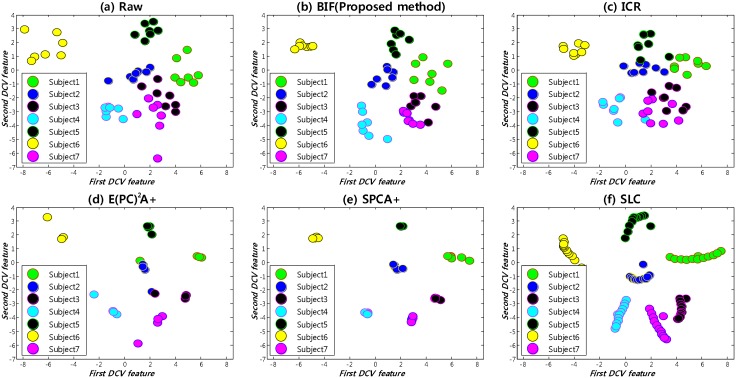
Sample distributions of 7 subjects that are generated from a single image in the two-dimensional discriminative common vector(DCV) feature space. (a) Raw. (b) BIF (Proposed method). (c) ICR. (d) E(PC^2^)A+. (e) SPCA+. (f) SLC.

We compared the proposed method to other methods dealing with the SSPP problem, namely ICR [15], E(PC^2^)A+ [14], SPCA+ [13] and SLC [17]. For 7 subjects in the CMU-PIE database—after generating images from a single image taken under a normal condition (frontal illumination) with several methods—we plotted the image samples in the two-dimensional discriminative common vector(DCV) feature space [4]. Compared with the distribution of the raw images in the CMU-PIE database (Fig 6a), the distribution of the images generated by the proposed method is most similar to that of the raw images, where the samples of the same subject are clustered closely and there is less overlap between samples for different subjects. Meanwhile, in the distributions of the other methods, some of the samples belonging to the same subject are widely scattered, and some samples belonging to different subjects are overlapped with each other (Fig 6c–6f).

### Face Recognition

To demonstrate how effective our proposed method is, we evaluated the performance of face recognition on the CMU-PIE, Yale B [28], 3D [29, 30], and Yale [31] databases. The characteristics of the different databases are presented in Table 2. For each database, we compared the face recognition performance of the above methods, which are ICR, E(PC^2^)A2+, SPCA+, and SLC. The center of each eye was manually detected in all of the images, and the subsequent horizontal alignment of the eyes was achieved with rotation, as in [32]. The cropping and rescaling of all of the face images meant that the central point of each eye could be statically positioned in an image measuring 80 (pixels) x 80 (pixels). Then, the histogram equalization process [32] was applied to the smaller-sized image. The one nearest-neighbor (NN) rule was used with the *l*_2_ norm as a classifier. As a feature extraction method for recognition, the DCV method was used for the SLC and the proposed method, while the PCA method was used for ICR, E(PC^2^)A2+, and SPCA+ because they are motivated by the PCA method.

**Table 2:** Characteristics of each database.

	CMU-PIE	Yale B	3D face	Yale
No. of subject	25	10	72	15
No. of images per subject	21	45	23	11
Illumination variation	large	large	large	small
Expression variation	none	none	none	large
Index of training images	11	1	1	6
No. of testing image	500	440	1584	150

For the evaluation of face recognition performance under illumination variation, we experimented on the CMU-PIE, Yale B, and 3D databases. We selected 65 of the 68 subjects in the CMU-PIE database, which have been placed under 21 illumination variations, as the other images were affected by defects or illumination-variation types were missing. Excluding the 40 subjects that were used to obtain the BIF, 25 subjects were used to evaluate the recognition rates. One image of each subject was selected from the images under frontal illumination (‘27_11′) for training, while the other 20 images were tested. There was no overlap of images between the training and testing sets. We used 45 face images of subjects in the frontal pose (YaleB/Pose00) from the Yale B database, which is comprised of 10 subjects placed under 64 illumination variations. To evaluate the recognition rates, one image of each subject under frontal illumination (*A* + 000*E* + 00) was selected for training, and the other images were used for testing. The 3D database consists of 106 subjects with 24 illumination variations, and each subject has two different sessions with an average of a 60-day gap. In this experiment, we selected 72 subjects with 23 illuminations in session 2 because some subjects were missed or did not include all types of illumination variations. One image of each subject under frontal illumination (‘*frame*1′) was selected for training, while the other 22 images were used for testing. We then conducted the experiments on the Yale database to evaluate face recognition performance for the various types of variations. The 15 subjects of the Yale database were placed under different illumination variations, and 165 gray images depict numerous facial expressions and the use or non-use of eyeglasses. Among them, one image of each subject labeled “*normal*” was selected for the training set, while the others were used as the test set.

The proposed method generated 6 virtual images with different illuminations for each subject. In ICR, the different numbers of synthesized images for each subject are generated by using the inter-class relationship depending on the database, as the number of nearest neighbors *k* are different in each database (*k* = 55, 46, 85, and 52 for CMU-PIE, Yale B, 3D, and Yale databases, respectively). The E(PC^2^)A+ method generated 3 images for training from the original image, which correspond to their 1/2-, first-, and second-order projected images, respectively. In SPCA+, 7 images for each subject were generated for training, which were obtained from different *n* order singular values, and in SLC, 11 images for each subject were added to the training set, which correspond to the symmetric images and linear combination virtual images.

**Fig 7:**
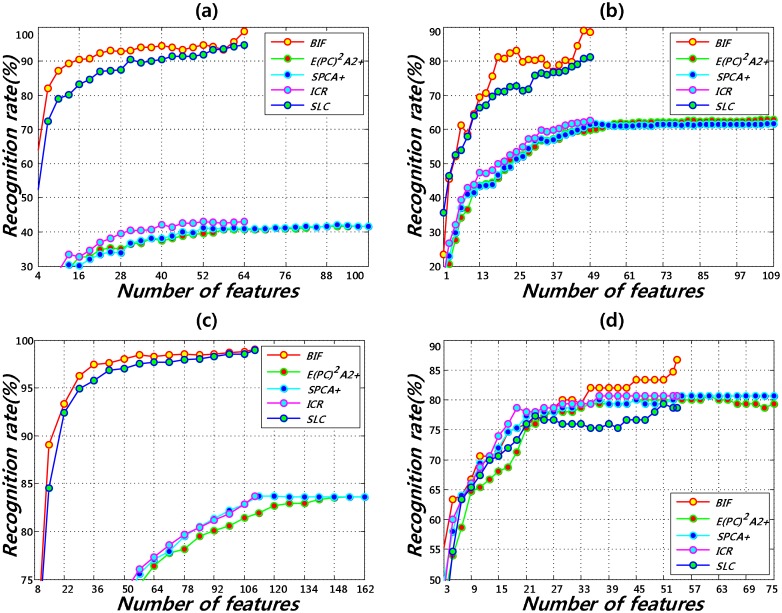
Face recognition results. (a) CMU-PIE database (b) Yale B database (c) 3D face database (d) Yale database.

Fig 7 shows that the proposed method outperformed the other methods for each database. For illumination variation, as shown in Fig 7a–7c, the proposed method gives a recognition rate of 99.00%, 88.86% and 99.05%, which are 4.00% ∼ 57.00%, 7.73% ∼ 27.27% and 0.06% ∼ 15.40% higher than the other methods for the CMU-PIE, Yale B, and 3D face databases, respectively. Similarly, as shown in Fig 7d, the proposed method outperforms the other methods with a recognition rate of 86.67% in the presence of different types of variations.

## Conclusions

The number of images for each subject is an important factor that affects the recognition performance when using appearance-based methods, which are widely used in face recognition. In face recognition practice, one frequently encounters the SSPP problem, i.e., a situation that only accessibility to a stored SSPP, which is an unfortunate reality underpinned by a number of key issues, including the difficulties associated with collecting samples and storage capability. In this paper, we propose a novel method to generate new images from a single image to address the SSPP problem. We extracted the BIF, which reflect the characteristics of various illumination conditions, and produced new images with six different illumination variations from a single image taken under frontal illumination. The experimental results showed that the generated images from the proposed method are distributed similarly to the real images that were taken under different illumination conditions. The proposed method therefore improved the face recognition performance compared with the other methods on the CMU-PIE, Yale B, Yale, and 3D face databases, all of which contain images with various types of variations.
